# Expression Analysis of SPARC/Osteonectin in Oral Squamous Cell Carcinoma Patients: From Saliva to Surgical Specimen

**DOI:** 10.1155/2013/736438

**Published:** 2013-12-16

**Authors:** Gabriella Aquino, Rocco Sabatino, Monica Cantile, Corrado Aversa, Franco Ionna, Gerardo Botti, Elvira La Mantia, Francesca Collina, Gabriella Malzone, Giuseppe Pannone, Nunzia Simona Losito, Renato Franco, Francesco Longo

**Affiliations:** ^1^Pathology Unit, Istituto Nazionale Tumori “Fondazione Pascale”-IRCCS, Napoli, Italy; ^2^Head and Neck Medical Oncology Unit, Istituto Nazionale Tumori “Fondazione Pascale”-IRCCS, Napoli, Italy; ^3^Department of Surgical Sciences, Section of Anatomic Pathology, Second Section of Oral Pathology, University of Foggia, Foggia, Italy

## Abstract

Oral squamous cell carcinoma (OSCC) remains a significant cause of morbidity and mortality, with approximately 540,000 new cases annually worldwide. The molecular mechanisms related to the pathogenesis of this disease are still poorly understood. The discovery of a molecular marker that allows the early detection of this cancer, which can be easily identified in biological samples, such as saliva, without intervening in advanced stages, is a challenge. Numerous studies have identified a panel of molecular markers differently expressed in OSCC and normal oral mucosa. In particular, it was found an aberrant expression of matricellular glycoprotein SPARC. SPARC is involved in normal tissue remodeling, regulating the deposition of extracellular matrix, but also in neoplastic transformation. In fact, aberrant SPARC expression was detected both in stromal cells associated with cancer and in tumor cells. The aim of our study was the evaluation of SPARC on a retrospective series of 119 OSCC cases and the validation of the obtained data on a prospective series of 27 patients with OSCC, of whom we have previously collected saliva, and smeared material. The obtained results were correlated with each other and with clinical pathological parameters at our disposal. The study demonstrated a prognostic value of SPARC, especially with regard to its expression in the stroma surrounding OSCC (*P* < 0.05).

## 1. Introduction

Squamous cell carcinoma (SCC) accounts for 90% of malignant tumors of the oral cavity. In particular, originating from oral and oropharyngeal cavity (OSCC and OPSCC, resp.) [1], it represents 4% of all malignancies in men and 2% in women. OSCC is characterized by high mortality, if not diagnosed in time, and significant percentages of full recovery if diagnosed in its early stages. Early diagnosis is therefore fundamental for prognostic definition and therapy. In fact, in maxillofacial surgery, every demolitive operation influences the vital functions of respiration, phonation, chewing, and swallowing and implies complicated and expensive technologies for reconstruction. It is therefore crucial to understand the molecular mechanisms related to the pathogenesis of this disease in order to designate new and more effective diagnostic and prognostic strategies. The main target is to identify new molecular markers that may be used in rapid and economic tests, which should be not invasive for OSCC patients.

Many studies, mostly carried out by gene-array technology, have identified a panel of molecular markers differentially expressed in the OSCC and in the normal oral mucosa [2, 3]. In particular, the gene expression of SPARC (secreted protein and rich in cysteine) has been showed deregulated in OSCC [4]. SPARC, also known as osteonectin or BM-40, is a glycoprotein belonging to a family of extracellular matrix proteins, whose function is to modulate cell-cell interactions and cell-matrix interaction [5]. SPARC acts as a key regulator of critical cellular functions such as proliferation, survival, and cell migration [6].

Although the role of SPARC is becoming increasingly evident in a variety of malignancies, there are conflicting informations about its contribution to tumor development and progression.

SPARC is differently expressed in various cancers and in the surrounding stroma compared to normal tissues, and its expression pattern is variable and highly dependent on the type of cancer. High levels of SPARC expression have been reported in breast [7, 8], prostate [9], colon rectal [10], and brain cancers [11, 12]. On the contrary, low levels of SPARC expression have been reported in other types of malignancies, as pancreas [13, 14], bladder cancer [15], and acute leukemia [16].

In our study, we proposed to analyze the expression of SPARC on a prognostic TMA, to verify if this protein could represent a potential new marker in OSCC, for noninvasive investigations.

In addition, samples from saliva, biopsy material, and fresh cell scrapings of patients with OSCC were also collected, and an analysis of gene expression by real-time RT-PCR was carried out. Using archival biopsies a prospective TMA was also built in order to evaluate the immunohistochemical expression of SPARC.

## 2. Material and Methods

### 2.1. Patients and Specimens

Histological blocks of cases have been selected in the files of Pathology Unit of National Cancer Institute Fondazione “G. Pascale” of Naples. All patients were Caucasians and all gave their written informed consent according to the institutional regulations.

This study was approved by the ethics committee of National Cancer Institute “G. Pascale” and our series included oral squamous cell carcinoma only. All cases were reviewed by two pathologists (Renato Franco and Nunzia Simona Losito) according to WHO classification criteria, using standard tissue sections and appropriate immunohistochemical slides. Medical records were reviewed for clinical information, and histologic parameters were determined from the H&E-stained slides. Clinicopathologic parameters evaluated for each tumor included patient age at initial diagnosis, tumor size, histologic grade, tumor stage, tumor recurrence or distant metastasis, anatomic tumor site, and deep invasion.

We have collected habits related to consumption of alcohol and smoking only for prospective series.

### 2.2. TMA Building

One hundred and nineteen OSCCs selected from 1998 to 2010, at the National Cancer Institute “Giovanni Pascale” of Naples, were used for building a first retrospective prognostic tissue microarray (TMA). Then, twenty-seven OSCC patients were selected for a second prospective TMA from 2011 to 2012.

Prognostic tissue microarray was built using two cores from different areas (a superficial one and one representative of the deep invasion) and, whenever possible, one core of normal mucosa of the same tissue block was arrayed for each case.

Prospective tissue microarray was built using only one representative core of OSCC.

All tumours and controls were reviewed by three experienced pathologists (Gerardo Botti, Nunzia Simona Losito, and Renato Franco). Discrepancies between pathologists for the same case were resolved in a joint analysis of the case. Tissue cylinders with a diameter of 1 mm were punched from morphologically representative tissue areas of each “donor” tissue block and brought into one recipient paraffin block (3 × 2.5 cm) using a semiautomated tissue arrayer (Galileo TMA). H&E staining of a 4 *μ*m TMA section was used to verify all samples.

### 2.3. SPARC TMA Based Immunostaining

Immunohistochemical staining on 4 *μ*m TMA serial sections was done carried out to evaluate the expression of SPARC marker in stromal and neoplastic cells. TMA slides were then deparaffinized in xylene and rehydrated through graded alcohols. Antigen retrieval was performed by microwave pretreatment in 0.01 M citrate buffer for 10 min. After protein block (BSA 5% in PBS 1x), the slides were incubated with primary antibody to Ki67 (DAKO Monoclonal Mouse Anti-Human Ki67 Ag Clone MIB-1 1 : 75) for 30 min and to human SPARC (dilution 1 : 1200, cod. ab55251, Abcam, Cambridge, UK) over night. The sections were rinsed in TBS and incubated for 20 min with Novocastra Biotinylated Secondary Antibody (RE7103), a biotin-coniugated secondary antibody formulation that recognized mouse and rabbit immunoglobulins. Then the sections were rinsed in TBS and incubated for 20 min with Novocastra Streptavidin-HRP (RE7104) and then peroxidase reactivity was visualized using a 3,3′-diaminobenzidine (DAB). Finally, sections were weakly counterstained with haematoxylin and coverslipped. Results were interpreted using a light microscope (Olympus BX53).

### 2.4. Evaluation of Ki67 and SPARC Immunostaining

SPARC and Ki67 stained tissue sections were evaluated by three pathologists (Renato Franco, Gerardo Botti, and Nunzia Simona Losito). For the proliferative index Ki-67 was defined as the percentage of immunoreactive tumor cells out of the total number of cells. The percentage of positive cells per case was scored according to 2 different groups: group 1: <50% (low proliferative activity) and group 2: >50% (high proliferative activity). In each sample we evaluated the percentage of both positive cancer cells and stromal cells surrounding tumor cells, counting the number of positive cells over the total cells in 10 nonoverlapping fields using ×400 magnification. For SPARC expression cytoplasmatic staining has been considered. Since there are not standardized criteria for SPARC staining evaluation, we have chosen to grade and score the extent of SPARC tumor and stromal immunostaining as follows: Score 0, negative staining; Score 1, 1–9%; Score 2, 10–100%.

### 2.5. RNA Extraction from Cellular Suspension and Paraffin Embedded Tissues

The sections obtained from paraffin-embedded samples were incubated at 37°C in the presence of xylene for 20 minutes approx. Total RNA was purified using High Pure FFPE RNA Micro Kit (Roche) following the manufacturer's instructions. Total RNA was isolated from saliva and scraping samples, using RNeasy Mini Kit (Qiagen GmbH, Hilden, Germany) following the manufacturer's instructions. All samples were treated with RNase-free DNase (Qiagen GmbH, Hilden, Germany) to prevent amplification of genomic DNA. A total of 1 *μ*g RNA was subjected to cDNA synthesis for 1 hr at 37°C using the Ready To Go You-Primer First-Strand Beads kit (Amersham Biosciences Europe Gmbh, Freiburg, Germany, cod. 27-9264-01) in a reaction mixture containing 0.5 *μ*g random hexamers (GeneAmp RNA PCR Random Hexamers Set N808-0127 Applied Biosystems, Foster City, CA).

### 2.6. Real-Time PCR

qRT-PCR was performed in a LightCycler system (Roche Molecular Biochemicals, Mannheim, Germany) using TaqMan analysis. In this system, all reactions were run in glass capillaries with the LightCycler TaqMan Master Mix (cod. 04735536001, Roche Molecular Biochemicals), in a volume of 20 *μ*L containing 2 *μ*L of cDNA and 1 *μ*L of specific TaqMan Gene Expression Assays for human *SPARC* (RealTime Designer Assay cod. 04162498001, Roche Molecular Biochemicals), according to the manufacturer's directions. All reactions were performed in triplicate. The thermal cycling conditions included a step of 20 sec at 95°C followed by a 40 cycles of 95°C for 1 sec and 60°C for 20 sec. The comparative *C*_*t*_ method was employed to determine the human *SPARC* gene variation, using as reference gene TaqMan Endogenous Controls Human *ACTB (*β*-actin)* Endogenous Control (RealTime Designer Assay cod. 05532957001, Roche Molecular Biochemicals). We identified a calibrator cell line that represents the unitary amount of the target of interest and consequently the samples express *n*-fold mRNA relative to the calibrator. Final amounts of target were determined as follows: target amount = 2_−*C*_*t*__, where *C*_*t*_ = [*C*_*t*_ (SPARC) −  *C*_*t*_ (ACTB)]_sample_  −  [*C*_*t*_ (SPARC)  −  *C*_*t*_ (ACTB)]_calibrator_.

### 2.7. Statistical Analysis

The association among SPARC and other clinicopathological parameters was determined using *χ* ^2^ and Student's *t*-tests.

Pearson's test was used to determine whether a relationship exists among the variables included in the study. The level of significance was defined as *P* < 0.05. All the statistical analysis were carried out using the Statistical Package for Social Science 13.0 software (SPSS Inc., Chicago, IL, USA).

## 3. Results

### 3.1. Clinic Pathological Features of OSCC Patients

The main clinical-pathological characteristics of the patients of the retrospective OSCC series are reported in Supplementary Table  1. (see Supplementary Material available online at http://dx.doi.org/10.1155/2013/736438).

This consisted of 119 patients aged between 31 and 92 years (mean age 70 years). 78 patients had lymph node metastases at diagnosis, the appearance of local recurrence was observed in 3 cases, and 1 patient had distant metastases. In addition, 29 patients were submitted to adjuvant chemotherapy, 60 to radiotherapy, and 27 to radio and chemotherapy. All selected patients were treated with chemotherapy after surgery and none of them had received the drug in the neoadjuvant therapy. Finally, 42 patients died over an average period of 24 months. The followup of 37 patients was not available. As regards histopathological grading, 20 cases were well-differentiated squamous cell carcinoma (G1), 66 squamous cell carcinomas were moderately differentiated (G2), and 33 cases were poorly differentiated carcinomas (G3). The tongue, with 71 cases, was the most affected location, followed by oral floor with 12 cases, lip with 3 cases, and other sites with 33 cases.

The prospective series (Supplementary Table 2) consisted of 27 patients aged between 44 and 84 years (mean age of 67 years). For the histopathological grading, 14 were moderately differentiated squamous cell carcinoma (G2), and 13 cases were poorly differentiated carcinomas (G3). The information test proposed to patients showed that (i) smoking patients were 16 out of 27, 12 of whom were heavy smokers (>20 cigarettes/die) and 4 light smokers (10–20 cigarettes/die); (ii) of the 27 patients, 17 consumed alcohol with such frequency: 1-2 cups, 12 out of 17 patients; 3-4 cups, 5 out of 17 patients (2 of whom were regular consumers of hard liquor); (iii) all 27 patients stated that they consume fruits and vegetables, with moderate (19 out of 27 patients) and high (8 out of 27 patients) frequency; (iv) patients have claimed to make dental visits, annually in 5 cases out of 27 and irregularly in 4 cases out of 27. 18 patients were never subjected to the dental visit.

### 3.2. IHC SPARC Expression on OSCC Patients Series

The morphological analyses aimed to reveal the subcellular localization and showed a cytoplasmic expression of SPARC. Moreover, SPARC was abundantly localized within the stroma adjacent to the tumor.

**Figure 1:**
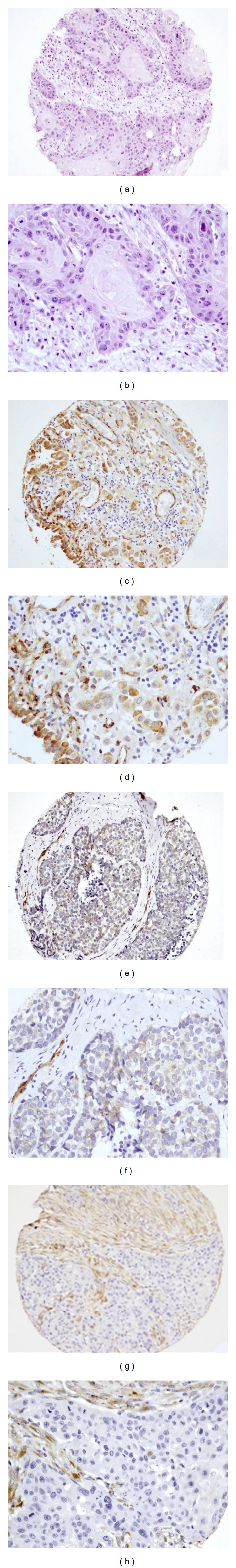
Representative hematoxylin/eosine and SPARC immunostaining on a prognostic oral squamous cell carcinoma (OSCC) TMA. (a, b) E/E (20x–40x); (c, d) high SPARC cytoplasmatic expression (20x–40x); (e, f) low SPARC cytoplasmatic expression (20x–40x); (g, h) negative SPARC tumoral expression in OSCC and strong SPARC immunostaining in stromal tissue (20x–40x).

The positivity of SPARC on the margin of the deep and superficial lesions and in the surrounding stroma were separately evaluated as shown in Figure 1 and schematized in Supplementary Figure 1.

On the deep margin of the lesion, immunohistochemical analysis of SPARC showed 21% of the cases with a moderate positivity and increased in 15% of cases, while 54% of cases were negative.

The expression of SPARC on the superficial margin of the tumor showed that 17% of the cases had a moderate positivity, 14% high positivity and 59% were negative. The analysis of the positivity of SPARC in the stroma adjacent to the deep side of the tumor revealed that 36% of the cases showed moderate positivity and 35.5% high positivity, whereas 18% were immunonegative. The analysis of the positivity of SPARC in the stroma adjacent to the superficial side of the tumor showed that 26% of the cases were moderately positive, 45% highly positive, and 18.5% showed a low positivity.

Regarding prospective OSCC patients series mean percentages of SPARC positivity evaluated in both superficial and deep lesions and in the surrounding stroma are summarized in Supplementary Figure 2.

The expression of SPARC in cancerous cells showed that 52% cases had a moderate positivity, 36% high positivity, and 11% cases resulted immunonegative.

The analysis of the positivity of SPARC in the stroma adjacent to the tumor revealed that 59% cases showed a moderate positivity and 36% high positivity.

Ki-67 expression analysis in the evaluable cases showed that 24% of the cases had a low positivity and 69% a high positivity.

### 3.3. Relation between SPARC Expression and Clinic Pathological Features and Survival in Retrospective OSCC Patients Series

All values of the immunohistochemical expression of SPARC on retroprospective series were crossed with classic clinicopathological parameters available.

The percentage of IHC tumor and stromal SPARC expression seemed to be directly related to each other (*P* < 0.05) (Supplementary Table 3).

In statistical elaboration, in which we proposed a single parameter that combined stromal and tumor expression, the expression of SPARC showed a significant correlation with histopathological grading (*P* = 0.011). In addition, a trend of statistical significance (*P* = 0.056) was highlighted as a result of correlation with the location of the primary lesion (Table 1). In fact, a high IHC expression of SPARC was observed in tumors of the tongue compared to those of the lips.

Moreover, a correlation between the expression of SPARC tumor on the deep margin of the lesion and the deep invasion was present (*P* = 0.016) (Table 2). Another significance was detected from the intersection between the stromal expression associated with deep margin of the lesion and the presence of metastases (*P* = 0.017) (Table 3).

Finally, other statistical elaborations, including SPARC superficial tumor expression and SPARC superficial stromal expression relation with clinicopathological parameters, are shown in Supplementary Tables 4 and 5.

**Figure 2:**
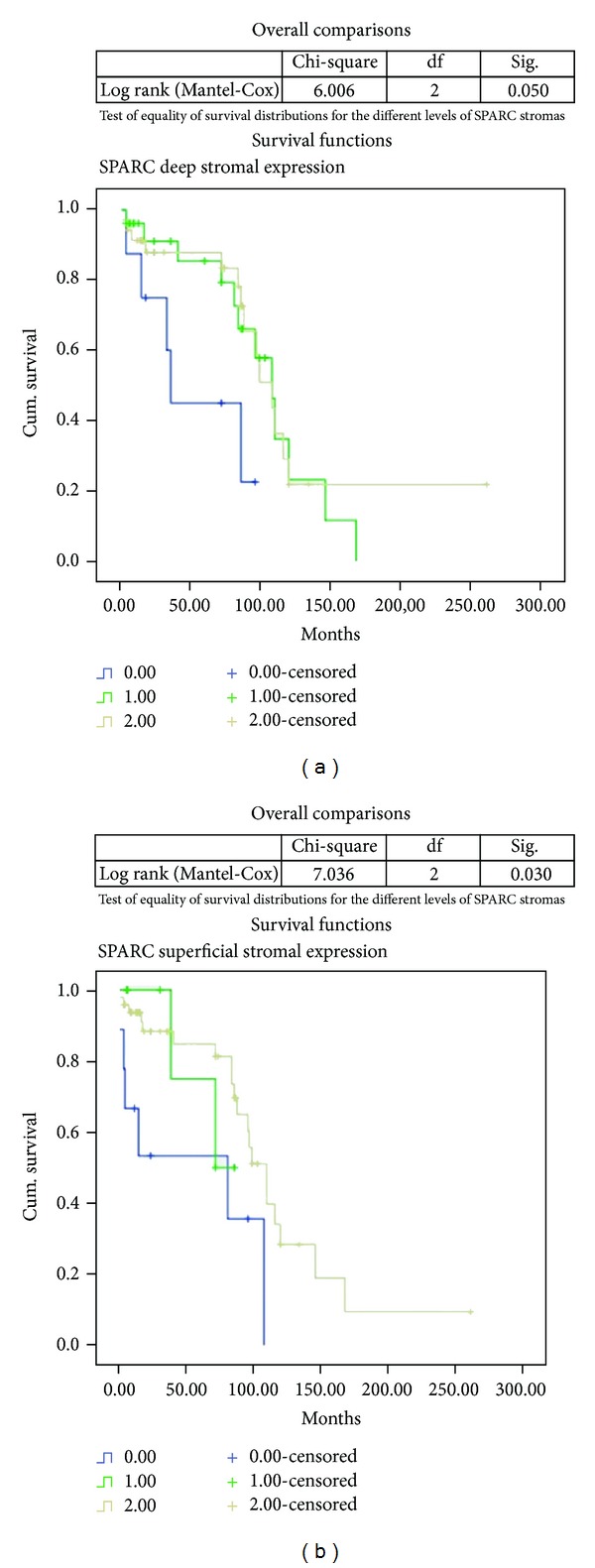
Kaplan-Meier curves related to SPARC expression and OSCC patients overall survival: (a) stromal deep expression; (b) stromal superficial expression.

The overall survival related to the expression of SPARC in stromal tissue at a deep and superficial level was very significant (*P* < 0.05) as shown in Figure 2.

**Figure 3:**
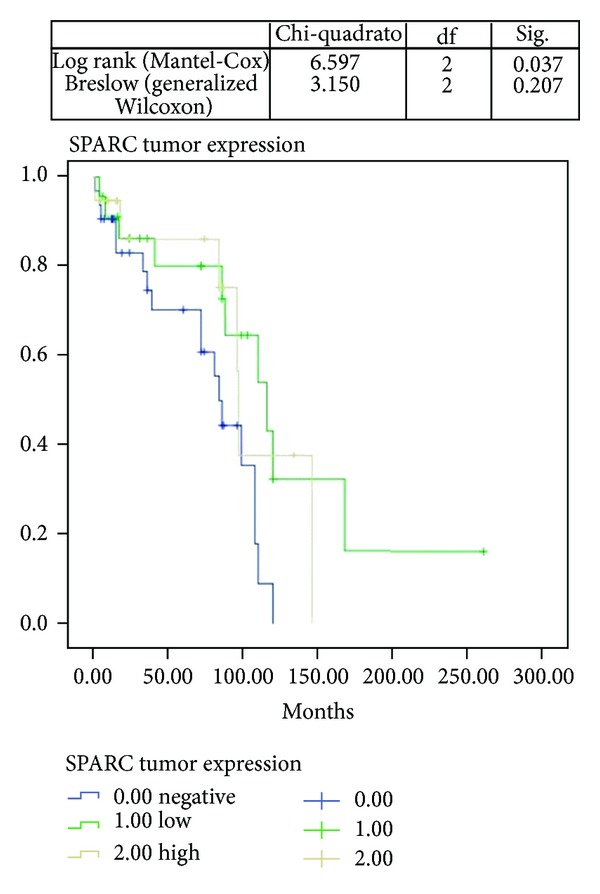
Kaplan-Meier curves related to SPARC tumoral expression and OSCC patients overall survival.

There also seemed to be a significant statistical correlation between the expression of SPARC in the tumor and the overall survival (*P* = 0.034) (Figure 3).

Therefore the Kaplan Meier's analysis highlighted that SPARC stromal and tumoral expression are a good prognosis marker.

### 3.4. Relation between SPARC Expression and Clinic Pathological Features of OSCC Prospective Series Patients

For the prospective series all values of immunohistochemical expression of SPARC were crossed with both classic clinic-pathological parameters and the information related to life habits. The statistical analysis showed a trend of correlation between high expression of SPARC in the tumor and high expression of SPARC in the stroma adjacent to the tumor (*P* = 0.082) (Table 4).

Moreover, the expression of stromal SPARC adjacent to the tumor showed a significant correlation with the high expression of Ki-67 (*P* = 0.040) and with the histopathologic grading (*P* = 0.025) (Table 4).

In addition, the correlation with medical records at our disposal showed a direct correlation between the expression of stromal SPARC and alcohol consumption (*P* = 0.005), as well as with the amount of cigarettes daily smoked (*P* = 0.006) (Table 5).

Finally a statistical elaboration between SPARC tumor expression and clinicopathological parameters highlighted a direct correlation with the amount of cigarettes daily smoked (*P* = 0.001) and with histopathological grading (*P* = 0.05). This statistical analysis is shown in Supplementary Table 6.

### 3.5. Expression Analysis of SPARC on Prospective OSCC Biological Samples

**Figure 4:**
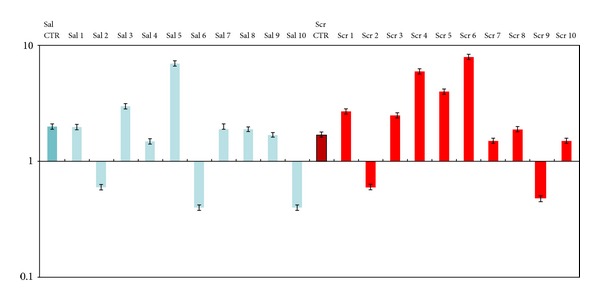
Real Time PCR gene expression analysis on prospective OSCC saliva and scraping samples. All reactions were performed in triplicate and data are expressed as mean of relative amount of mRNAs levels.

The analysis of gene expression for SPARC was performed on 10 samples of saliva and scraping taken from the patients included in the prospective series (Figure 4). In particular, three samples (2, 6, 10) showed a silent expression of SPARC gene, while samples 1, 3, 5, 7 had a moderate expression of SPARC (value between 1- and 10-fold increase) compared to normal.

**Figure 5:**
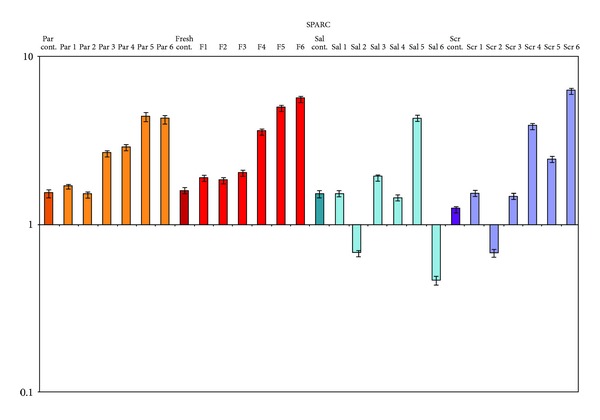
Real Time PCR gene expression analysis on prospective OSCC fresh and archival samples. All reactions were performed in triplicate and data are expressed as mean of relative amount of mRNAs levels.

The same analysis was also performed on 6 selected samples from prospective series with a different IHC expression of SPARC protein for which we compared in parallel SPARC expression on paraffin tissues, cryostored fresh tissues, saliva, and scraping (Figure 5).

The results of the analysis carried out on samples of saliva and scraping showed an increased expression of SPARC in 20% of saliva samples and in 30% of scraping samples, compared to normal controls. The gene expression of SPARC on the cryostored and archival specimens of the same patients, however, showed higher values, comparable to those revealed by immunohistochemistry (values between 5- and 10-fold increase).

## 4. Discussion

Squamous cell carcinoma of the oral cavity is one of the most common malignancies in the population, but its diagnosis is often late for the body location in which it occurs and for the irregularity with which patients yet consult specialists [17]. The diagnostic investigations for this tumor are currently very invasive for patients, as the biopsy requires the removal of tissue at sites often highly sensitive. For this reason, easier diagnostic strategies, which would allow stratifying for biopsy for only patients at risk, should be investigated. The biological material, such as scraping and saliva, would represent an ideal system for the identification of overexpressed and specific markers [18, 19]. However, to date, a molecular marker, whose sensitivity can give the certainty either of the presence or the risk of this cancer, has not yet been identified.

Gene profiling studies, showed the different expression of many genes between normal mucosa and oral cavity tumors. It has been consistently reported the aberrant expression of SPARC/osteonectin, identifying between several panels of genes [20, 21].

For this reason we proposed to verify the altered expression of SPARC in OSCC by setting up a prognostic TMA to correlate the expression of this marker not only with classic clinic pathological features, but also to analyze in more detail its subcellular localization.

Analyzed data on this first TMA, consisting of 119 tissue specimens of patients with OSCC, have shown that the overexpression of SPARC on the deep side of the lesion correlates with deep invasion. We also found a trend of association between the deep invasion and the overexpression of SPARC in stromal cells of the deep layer. In addition, SPARC was less expressed in tumors localized at the lip, which appear to be the least aggressive.

The data associated with the expression of SPARC in the stroma was particularly interesting for several reasons: (i) its expression significantly correlated with the presence of metastases; (ii) its expression in the deep margin very significantly correlated with patients survival; (iii) its expression significantly correlated with tumor grade. These data allowed us not only to confirm the prognostic role of this marker in OSCC, but also to highlight its aberrant activity particularly in the stromal cells surrounding the deep tumor.

Another aim of the study was to determine whether the gene expression of this marker could be detected in biological samples such as saliva and scraping. Thus, we have selected another prospective case series of samples on which we have carried out not only the molecular analysis by Real Time RT-PCR, but also the immunohistochemical expression of SPARC from archival corresponding samples. All samples, of which we had the opportunity to compare saliva, scraping, fresh tissue, and paraffin, showed a trend of increased expression of SPARC compared with normal controls, but this expression did not correspond properly to the protein expression detected by IHC on archival samples. This could depend on both the quality and quantity of the purified RNA, from biological samples of scraping and saliva, but also by the location of SPARC, which was prevalent in the deep side of the surrounding stroma. This, of course, did not allow us to obtain similar results, because the biological material was mainly composed of neoplastic cells from the surface layer of the tumor. This hypothesis was further confirmed by IHC carried out on the same samples of the prospective series. The absence of expression of SPARC in the superficial component of the tumor might also suggest that a significant increase in expression is mainly observed in tumor stages more locally advanced. On this last series it was possible not only to confirm the data of aberrant expression of SPARC in the stroma of the deep layer with a significance even more evident, but also to correlate the high expression of SPARC with the grade of the tumor and proliferation index.

For these patients the expression of SPARC was also correlated with the habits of consumption of alcohol and smoking. Statistical elaboration related to stromal SPARC expression was significantly associated with habitual smokers and consumers of alcohol, highlighting how these habits can be, once again, poor prognostic factors for this cancer [22–24].

In conclusion, our data have allowed us not only to establish the prognostic value of SPARC in squamous cell carcinoma of the oral cavity, but also to highlight that the main activity of this protein is carried out predominantly in the tumor microenvironment, being its aberrant expression manifested/exhibited in stromal cells surrounding the tumor [25]. However, selected patients are very heterogeneous mainly with regard to staging and therapies employed; thus, the correlation with prognosis has to be well pondered. Morphological analysis have also allowed better determining the cellular localization of this protein in the deep side of the tumor, and this can explain why in biological samples, such as scraping and saliva, it was not possible to determine the overexpression of this marker.

However, since the identification of a rapid, inexpensive, and noninvasive test represents one of the major challenges in OSCC diagnosis, a great effort must be made from basic research to identify new and more reliable molecular targets.

## Supplementary Material

Supplementary Table 1 : Main clinical features of the patients arranged in the retrospective TMA.
Supplementary Table 2 : Main clinical features of the patients arranged in the prospective TMA.
Supplementary Table 3: Significant correlation between SPARC stromal and tumor expression.
Supplementary Table 4 : Correlation between SPARC superficial tumor expression and main clinical features of the patients arranged in the retrospective TMA.
Supplementary Table 5 : Correlation between SPARC superficial stromal expression and main clinical features of the patients arranged in retrospective TMA.
Supplementary Table 6 : Prospective OSCC patients correlation between SPARC tumor expression and main clinical features, and between SPARC stromal expression and personal habits.
Supplementary Figure 1 : Percentage distribution of SPARC differential expression in the prospective TMA.
Supplementary Figure 1 : Percentage distribution of SPARC differential expression in the prospective TMA.

## Figures and Tables

**Table 1:** Relation between SPARC and clinic pathological features of retrospective OSCC patients series.

SPARC	Grading			Site			Deep invasion		*T*		*N*			Sex		Age		
	1	2	3	Other	Toungue	Lips	<10 mm	>10 mm	*T*1\2	>*T*2	0	1	2	M	F	<40	41–55	>56
Negative	14	17	8	10	27	2	18	21	23	16	20	10	9	30	9	1	3	35
Positive	7	39	17	37	36	0	24	39	39	24	29	11	23	47	16	2	2	59

Pearson chi-square	0.011			0.056			0.42		0.76		0.31			0.71		0.58		

**Table 2:** Relation between SPARC deep tumor expression and clinic pathological features of retrospective OSCC patients series.

SPARC deep tumour expression	Grading			Anatomical site		Deep invasion		*T*		*N*			Age		Sex	
	1	2	3	Tongue	Other	<10 mm	>10 mm	*T*1\2	>*T*2	0	1	2	<50	>51	F	M
0	9	25	10	21	23	24	20	24	20	20	5	19	2	42	12	32
1	5	10	4	6	12	3	16	11	8	8	6	5	2	19	6	13
2	5	21	10	16	19	17	19	27	9	18	9	9	1	36	6	30

Pearson chi-square	0.8			0.57		0.016		0.152		0.21			0.87		0.38	

**Table 3:** Relation between SPARC deep stromal expression and clinic pathological features of retrospective OSCC patients series.

SPARC deep stromal expression	Grading			Anatomical site		Deep invasion		*T*		*N*			Age		Sex	
	1	2	3	Tongue	Other	<10 mm	>10 mm	*T*0\1	>*T*2	0	1	2	<50	>51	F	M
0	3	6	2	5	6	7	4	7	4	8	0	3	1	12	3	8
1	8	18	11	14	21	11	26	22	15	11	13	13	2	37	7	30
2	8	32	10	22	28	24	26	33	17	28	7	15	2	48	14	36

Pearson chi-square	0.620			0.91		0.079		0.822		0.017			0.65		0.606	

**Table 4:** Relation between SPARC stromal expression and clinic pathological features of prospective OSCC patient series.

SPARC stromal expression	Grading			Anatomical site		Ki 67 expression		*T*		Sparc tumor expression		Age		Sex	
	1	2	3	Other	Tongue	Low	High	*T*1\2	>*T*2	Negative	Positive	<50	>51	F	M
Negative	0	8	2	6	4	0	7	9	1	6	5	1	9	3	7
Positive	0	4	10	7	7	6	8	13	1	3	11	1	13	9	5

Pearson chi-square	0.025			0.45		0.040		0.8		0.08		0.5		0.13	

**Table 5:** Relation between SPARC stromal expression and personal habits of prospective OSCC patients series.

SPARC stromal expression	Fruits and vegetables consumption			Smokers		Alcohol consumption		
	No	Low	High	Light	Heavy	No	Low	High
Negative	0	8	2	3	2	3	7	0
Positive	1	8	5	0	10	5	4	5

Pearson chi-square	0.43			0.006		0.05		
