# Supplementary material for: Expression Analysis of SPARC/Osteonectin in Oral Squamous Cell Carcinoma Patients: From Saliva to Surgical Specimen
Source: Biomed Res Int. 2013 Dec 16;2013:736438. doi: 10.1155/2013/736438 (PMC3876772; doi:10.1155/2013/736438)
Supplement: Supplementary file 1 — Supplementary Table 1 : Main clinical features of the patients arranged in the retrospective TMA. Supplementary Table 2 : Main clinical features of the patients arranged in the prospective TMA. Supplementary Table 3: Significant correlation between SPARC stromal and tumor expression. Supplementary Table 4 : Correlation between SPARC superficial tumor expression and main clinical features of the patients arranged in the retrospective TMA. Supplementary Table 5 : Correlation between SPARC superficial stromal expression and main clinical features of the patients arranged in retrospective TMA. Supplementary Table 6 : Prospective OSCC patients correlation between SPARC tumor expression and main clinical features, and between SPARC stromal expression and personal habits. Supplementary Figure 1 : Percentage distribution of SPARC differential expression in the prospective TMA. Supplementary Figure 1 : Percentage distribution of SPARC differential expression in the prospective TMA. [file 736438.f1.docx]

|  | **Overall Population (119)** |
| --- | --- |
| **Age at the diagnosis** |  |
| **<45** | 4\119 |
| **>45** | 115\119 |
|  |  |
| **Gender** |  |
| **M** | 83\119 |
| **F** | 36\119 |
|  |  |
| **Grading** |  |
| **G1** | 20\119 |
| **G2** | 66\119 |
| **G3** | 33\119 |
|  |  |
| **Overall free disease survival** | 40\85 |
| **Death** | 42\85 |
| **No follow-up** | 37\119 |
| **Recurrence** | 3\85 |
|  |  |
| **Tumor staging** |  |
| **T1-2** | 75\119 |
| **T3-4** | 44\119 |
|  |  |
| **Lymph Node metastases** | 78\119 |
| **Distant metastases** | 1\119 |
| **No metastasis** | 40\119 |
|  |  |
| **THERAPY** |  |
| **Chem** | 29\89 |
| **RxT** | 60\89 |
| **Chem+RxT** | 27\89 |
| **No Therapy information** | 20\119 |
|  |  |
| **Primary anatomical Site** |  |
| **Tongue** | 71\119 |
| **Other sites** | 45\119 |
| **Lips** | 3\119 |

**Supplementary Table 1**

|  | **Overall Population (27)** |
| --- | --- |
| **Age** |  |
| **<50** | 2\27 |
| **>50** | 25\27 |
|  |  |
| **Gender** |  |
| **M** | 15\27 |
| **F** | 12\27 |
|  |  |
| **Grading** |  |
| **G1** | 0\27 |
| **G2** | 14\27 |
| **G3** | 13\27 |
|  |  |
| **Tumor stage** |  |
| **T1-2** | 25\27 |
| **T3-4** | 2\27 |
|  |  |
| **Smokers** | 16\27 |
| **Heavy smokers (>20sig)** | 12\10 |
| **Light smokers (< 19 sig)** | 4\10 |
|  |  |
|  |  |
| **Drinkers** | 17\27 |
| **1-2 alcool cups daily assumption** | 12\17 |
| **3-4 alcool cups daily assumption** | 5\17 |
|  |  |
|  |  |
| **Fruits/Vegetables** |  |
| **Moderate** | 19\27 |
| **Hight** | 8\27 |
|  |  |

**Supplementary Table 2**

|  | SPARC  Tumor Expression | |
| --- | --- | --- |
| SPARC Stromal  Expression | **Negative** | **Positive** |
| Negative | 15 | 0 |
| Positive | 28 | 59 |
| *Pearson*  *Chi-square* | ,000 | |

**Supplementary Table 3**

|  | Grading | | | Anatomical site | | Deep Invasion | | T | | | N | | | Age | | SEX | |
| --- | --- | --- | --- | --- | --- | --- | --- | --- | --- | --- | --- | --- | --- | --- | --- | --- | --- |
| SPARC Superficial  Tumour Expression | **1** | **2** | **3** | **Tongue** | **Other** | **< 10mm** | **> 10mm** | **T1\2** |  | **>T2** | **0** | **1** | **2** | **<50** | **>51** | **F** | **M** |
|  |  |  |  |  |  |  |  |  |  | |  |  |  |  |  |  |  |
| 0 | 10 | 23 | 7 | 18 | 22 | 18 | 22 | 23 |  | 17 | 19 | 7 | 14 | 2 | 40 | 11 | 29 |
| 1 | 4 | 15 | 5 | 11 | 12 | 6 | 18 | 15 |  | 9 | 13 | 7 | 4 | 2 | 27 | 6 | 18 |
| 2 | 3 | 12 | 10 | 10 | 14 | 12 | 13 | 16 |  | 9 | 11 | 6 | 8 | 1 | 30 | 4 | 21 |
|  |  |  |  |  |  |  |  |  |  |  |  |  |  |  |  |  |  |
| Pearson *Chi-square* | ,258 | | | ,91 | | ,191 | | ,853 | | | ,552 | | | ,368 | | ,559 | |

**Supplementary Table 4**

|  | Grading | | | Anatomical site | | Deep Invasion | | T | | N | | | Age | | SEX | |
| --- | --- | --- | --- | --- | --- | --- | --- | --- | --- | --- | --- | --- | --- | --- | --- | --- |
| SPARC Superficial  Stromal  Expression | **1** | **2** | **3** | **Tongue** | **Other** | **< 10mm** | **> 10mm** | **T1\2** | **>T2** | **0** | **1** | **2** | **<50** | **>51** | **F** | **M** |
|  |  |  |  |  |  |  |  |  |  |  |  |  |  |  |  |  |
| 0 | 3 | 5 | 4 | 7 | 5 | 7 | 5 | 6 | 6 | 7 | 2 | 3 | 2 | 13 | 5 | 7 |
| 1 | 12 | 21 | 9 | 17 | 23 | 13 | 29 | 24 | 18 | 17 | 10 | 15 | 2 | 42 | 9 | 33 |
| 2 | 2 | 25 | 10 | 16 | 21 | 16 | 21 | 25 | 12 | 19 | 8 | 10 | 1 | 42 | 8 | 29 |
|  |  | | |  | |  | |  | |  | | |  | |  | |
| Pearson *Chi-square* | **,087** | | | ,604 | | ,191 | | ,46 | | ,552 | | | ,368 | | ,31 | |

**Supplementary Table 5**

|  | Grading | | | Anatomical site | | Ki 67 expression | | T | | Age | | SEX | |
| --- | --- | --- | --- | --- | --- | --- | --- | --- | --- | --- | --- | --- | --- |
| SPARC  tumoral Expression | **1** | **2** | **3** | **Tongue** | **Other** | **low** | **high** | **T1\2** | **>T2** | **<50** | **>51** | **M** | **F** |
|  |  |  |  |  |  |  |  |  |  |  |  |  |  |
| Negative | 0 | 7 | 2 | 5 | 4 | 1 | 6 | 8 | 1 | 1 | 8 | 6 | 3 |
| Positive | 0 | 6 | 10 | 7 | 9 | 5 | 9 | 14 | 1 | 2 | 14 | 8 | 8 |
| Pearson *Chi-square* | **,053** | | | ,57 | | ,30 | | ,7 | | ,91 | | ,4 | |

|  | Fruits and Vegetables  consumption | | | Smokers | | Alcohol consumption | | |
| --- | --- | --- | --- | --- | --- | --- | --- | --- |
| SPARC  Stromal Expression | **No** | **Low** | **High** | **light** | **Hevvy** | **No** | **low** | **high** |
|  |  |  |  |  |  |  |  |  |
| Negative | 0 | 7 | 2 | 4 | 1 | 2 | 6 | 1 |
| Positive | 1 | 9 | 5 | 0 | 11 | 6 | 6 | 4 |
| Pearson *Chi-square* | ,43 | | | ,001 | | ,37 | | |

**Supplementary Table 6**

**
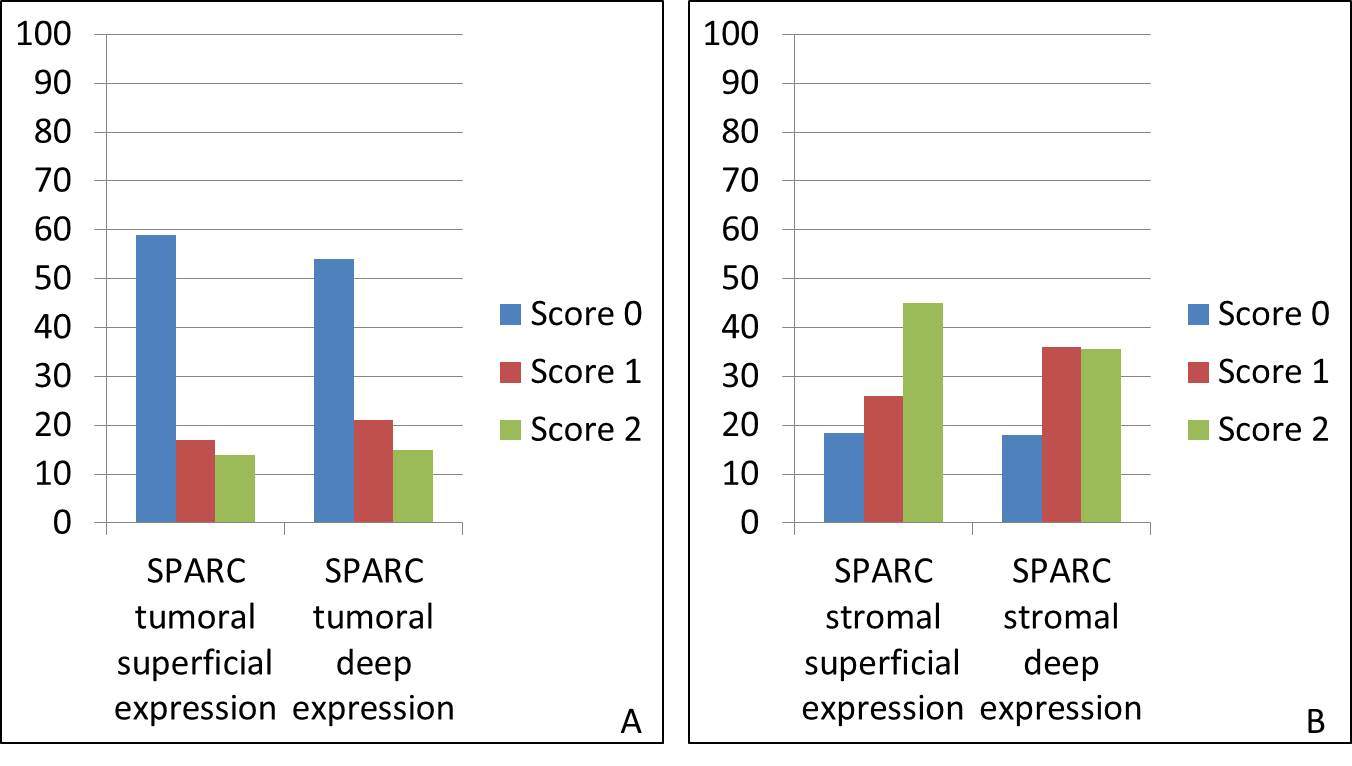
Supplementary Figure 1**

**
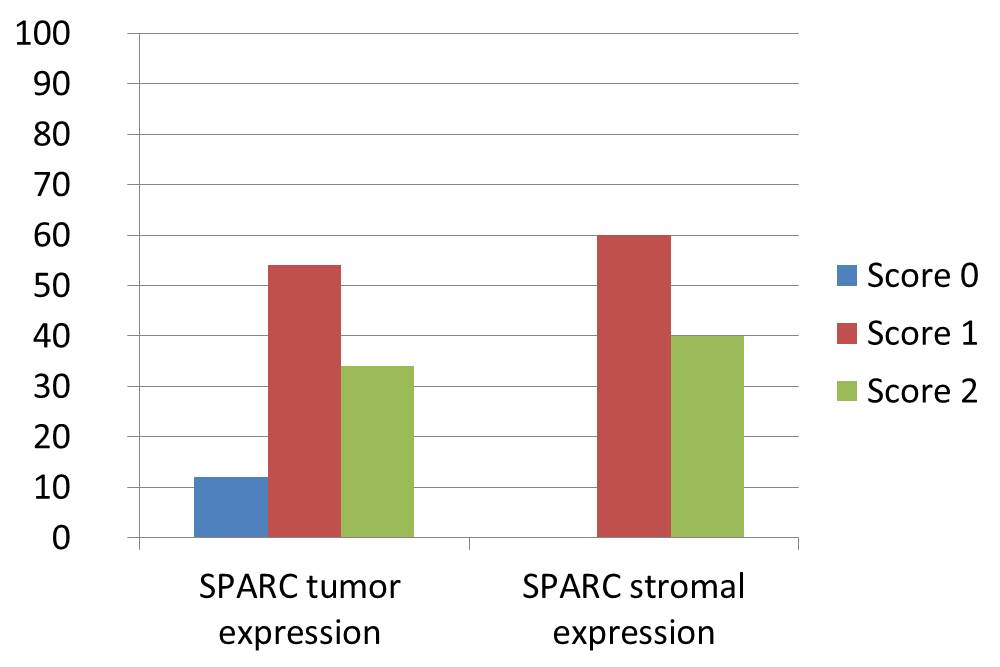
Supplementary Figure 2**
